# Retraction: Effect of bimetal element doping on the low-temperature activity of manganese-based catalysts for NH_3_-SCR

**DOI:** 10.3389/fchem.2023.1208136

**Published:** 2023-05-09

**Authors:** Frontiers Editorial Office

The journal retracts the 22 July 2022 article cited above.

Following concerns regarding the originality of the article, an investigation was conducted in accordance with Frontiers’ policies. It was found that the article should be retracted, because of an unacceptable level of similarity to the Royal Society of Chemistry Advances article “Selective catalytic reduction of NO_x_ by low-temperature NH_3_ over Mn_x_Zr_1_ mixed-oxide catalysts” - https://pubs.rsc.org/en/Content/ArticleLanding/2022/RA/D1RA08800A. Following the Committee on Publication Ethics guidelines, this article, published most recently, is retracted.

This retraction was approved by the Chief Editors of Frontiers in Chemistry and the Chief Executive Editor of Frontiers. The authors do not agree to correspondence regarding this retraction.

